# Human Immunodeficiencies Related to Defective APC/T Cell Interaction

**DOI:** 10.3389/fimmu.2015.00433

**Published:** 2015-08-28

**Authors:** Marinos Kallikourdis, Antonella Viola, Federica Benvenuti

**Affiliations:** ^1^Humanitas University, Rozzano, Italy; ^2^Adaptive Immunity Laboratory, Humanitas Clinical and Research Center, Rozzano, Italy; ^3^Venetian Institute of Molecular Medicine, Padova, Italy; ^4^Cellular Immunology, International Centre for Genetic Engineering and Biotechnology, Trieste, Italy

**Keywords:** immune synapse, immunodeficiencies, actin cytoskeleton, chemokines, T cell activation

## Abstract

The primary event for initiating adaptive immune responses is the encounter between T lymphocytes and antigen presenting cells (APCs) in the T cell area of secondary lymphoid organs and the formation of highly organized intercellular junctions referred to as immune synapses (IS). *In vivo* live-cell imaging of APC–T cell interactions combined to functional studies unveiled that T cell fate is dictated, in large part, by the stability of the initial contact. Immune cell interaction is equally important during delivery of T cell help to B cells and for the killing of target cells by cytotoxic T cells and NK cells. The critical role of contact dynamics and synapse stability on the immune response is well illustrated by human immune deficiencies in which disease pathogenesis is linked to altered adhesion or defective cross-talk between the synaptic partners. The Wiskott–Aldrich syndrome (WAS) is a severe primary immunodeficiency caused by mutations in the Wiskott–Aldrich syndrome protein (WASp), a scaffold that promotes actin polymerization and links TCR stimulation to T cell activation. Absence or mutations in WASp affects intercellular APC–T cell communications by interfering with multiple mechanisms on both sides of the IS. The warts, hypogammaglobulinemia, infections, and myelokathexis (WHIM) syndrome is caused by mutations in CXCR4, a chemokine receptor that in mutant form leads to impairment of APC–T cell interactions. Present evidences suggest that other recently characterized primary immune deficiencies caused by mutation in genes linked to actin cytoskeletal reorganization, such as WIP and DOCK8, may also depend on altered synapse stability. Here, we will discuss in details the mechanisms of disturbed APC–T cell interactions in WAS and WHIM. Moreover, we will summarize the evidence pointing to a compromised conjugate formation in WIP, DOCK8, and X-linked lymphoproliferative syndrome.

## Introduction

Cells of the immune system communicate with one another by physical contacts and by soluble signals that may act on the interacting cells or at a distance. The formation of intercellular junctions is essential to bring receptor–ligand couples close enough to trigger downstream signaling and to transmit activatory/inhibitory signals between the two cells. Tight membrane apposition is also a prerequisite to allow focused delivery of soluble factors in a spatially confined fashion, ensuring specificity and effectiveness during killing of targets or polarized secretion of soluble mediators. Immune cell interaction is supported by several interconnected systems that assist the various stages of contact formation from initial scouting to adhesion and stabilization of the junction. The importance of such pathways is underscored by human pathologies caused by mutations in genes controlling these systems.

## APC–T Cell Encounter in the T Cell Area of Lymph Nodes

The first challenge for a T cell entering the T cell area of a lymph node is to find its cognate antigen on the surface of a dendritic cell (DC). This process is aided by the strategic distribution of DCs in an extensive network and by chemokine cues that guide motility and positioning in lymph nodes. *In vivo* imaging experiments have shown that lymphocytes entering the T-cell zones move randomly over densely packed networks of DCs and fibroblastic reticular cells (FRCs) (1, 2). This motility is driven by CCR7-binding chemokines. Besides CCL21, other chemokines produced in lymph nodes may coordinate specific encounters between cells. Thus, CCL3 and CCL4 seem to be involved in recruitment of naïve CD8^+^ T cells, which can upregulate CCR5 expression during inflammation, to sites where they can receive help from CD4^+^ T cells (3). CXCR3 expression on CD4^+^T cells is important for the interaction with antigen bearing DCs and for the global intranodal positioning of T cells (4). Moreover, the same chemokine receptor selectively controls repositioning of memory T cells within lymph nodes during a recall response (5).

Interaction of the TCR with cognate antigen results in the activation of phospholipase C-γ and Ca^2+^ influx via calcium release activated channels (CRAC) Orai1/CRACM1 in the plasma membrane (6, 7). Among the other effects, Ca^2+^ influx induces ATP synthesis and release (8) that, in turns, induces P2X4/P2X7-mediated calcium waves in the neighboring lymphocytes and acts as a paracrine signaling molecule that regulates T cell motility during immune responses (9). ATP-induced Ca^2+^ waves induce a “stop” not only in cells that have already found their antigenic partners but also in lymphocytes that may be potentially triggered within the tissue. Several studies have indeed observed that in the lymph node microenvironment there is a significant drop in the velocity of polyclonal T cells during antigenic stimulation of TCR-specific cells (10, 11). The reduced motility of T lymphocytes in a tissue where antigenic recognition is occurring may be strategic for a better scanning of resident DCs and, in this perspective, extracellular ATP may alter the equilibrium between adhesive and chemoattractant forces operating in lymph nodes during T cell priming and thus modify T cell activation. Interestingly, destabilization of T–DCs conjugates *in vivo* by regulatory T cells is, in part, due to high levels of expression of CD39 and CD73, two cell surface ecto-enzymes that hydrolyze extracellular ATP to ADP, AMP and adenosine that, acting through the A2A receptor, prevents activation and proliferation of CD4^+^ T cells (12, 13).

## The Duration of APC–T Cell Contacts and the Consequences for T Cell Activation

The dynamics of cellular contacts and the functional consequences of short and prolonged cellular interactions in terms of T cell activation have been investigated mostly in the context of naïve T cells priming by DCs. *In vitro* studies showed that T cells remain stably attached to DCs in conditions that lead to T cell activation, whereas short intermittent contacts dominate when DCs are immature and unable to induce activation. With the limits of an *in vitro* analysis, these findings provided one of the first correlations between contact duration and function (14). An opposite result, i.e., short contacts may be enough to trigger naïve T cell activation, was obtained when analyzing cells in a collagen 3D matrix, suggesting that the requirements for T cell activation may depend on the context (15). Direct imaging of the immune response in lymph nodes revealed the presence of both sequential, brief, T–DC contacts (kynapses) and long antigen-specific contacts (synapses) (16). Different phases of short- and long-lasting antigen presenting cell (APC)–T contacts alternates during initial priming and longer arrest of T cells on the APC surface predominates in conditions of full T cell activation (17–19). This concept was later refined by studies showing that the affinity of the pMHC for the TCR critically determines contact duration. High-affinity antigens induce a complete T cell stop, whereas low-affinity antigens cause only T cell deceleration (20–22). Interestingly, the presence of bystander cells, such as regulatory T cells, modifies contact dynamics hampering the formation of stable contacts (12, 20). The state of T cell activation is a further critical parameter that determines contact dynamics. Naïve T cells stop and form mostly synapses upon antigen recognition, whereas previously activated T cells can collect activatory signals from kinapses (23). It has also emerged that kinapses may lead to T cell activation when antigen density is high enough to allow integration of signals over multiple serial encounters (24).

## Molecular Structure of the Immune Synapse

**Figure 1:**
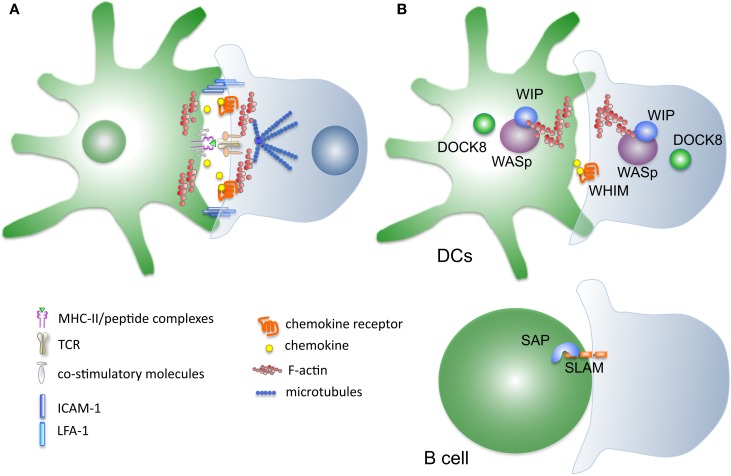
**Mutations in proteins controlling synapse stability linked to development of primary immune deficiencies**. **(A)** Schematic representation of the molecules implicated DC–T cells cross-talk during synapse formation. Several interconnected systems, including membrane receptors and cytosolic proteins, contribute to form and stabilize the interaction between the two cell types. **(B)** Disease-causing mutations targeting proteins that control synapse stability. Wiskott–Aldrich syndrome protein WASp, causative of WAS syndrome, is a key node controlling actin polymerization in immune cells. Mutations in WASp have been associated to several defects in synapse formation on both the T-cell and the DC side. Most recently discovered mutations in DOCK8 and WIP cytoskeletal regulators cause immunodeficiency syndromes whose cellular basis include disturbance of the intercellular interactions. SAP, an adaptor for SLAM receptors mutated in XLP syndrome, is affecting selectively the stability of the B–T synapse.

*Ex-vivo* analysis of single T cells engaged in contact with APCs has been instrumental to understand the subcellular reorganization occurring in T cells during activation (Figure 1). Because of some analogies with the mode of intercellular communication used by neurons, the specialized structure formed between a T cell and an antigen presenting B cell was named as “immune synapse” (25, 26). The immune synapse (IS) was initially described as a central area containing signaling components such as the TCR and PKC-θ kinase (cSMAC), a peripheral ring containing adhesive molecules (pSMAC), and a distal region rich in actin (dSMAC). Subsequent studies using planar bilayer as surrogate APCs allowed the quantitative and dynamic analysis of synapse formation in T cells and to assess the contribution of single receptors (MHC peptide complexes, adhesion, and co-stimulatory molecules) on the reorganization of signaling platforms, cytoskeletal remodeling, and polarized vesicular trafficking (Figure 1). These studies revealed that microclusters (MCs) of 10–20 TCRs molecules forms in the dSMAC and are translocated into the cSMAC, where the signaling activity of the TCR extinguishes [(27–30) and reviewed in Ref. (31)]. An essential component to coordinate TCR signaling is the actin cytoskeleton. This is needed to support early events of T cell activation, such as clustering of TCRs in MCs, recruitment of signaling complexes to MC, and later mobility of signaling platforms. In turn, recruited signaling molecules, such as the adaptor LAT, serve as platforms to dock cytoskeletal regulatory proteins, such as Vav and Wiskott–Aldrich syndrome protein (WASp), necessary to sustain T cell activation (30, 32). Most recently, a novel view of the actin cytoskeleton as a global regulator of the cytoplasm poroelasticity and consequently of T cell signaling is emerging (33).

Interconnected to the role of actin cytoskeleton, the integrin LFA-1, acts at several levels in the IS. First of all, lateral movements of LFA-1, ensured by linkage with the underlying actin cytoskeleton, are essential to ensure correct T cell activation (31, 34). Besides its function in supporting synaptic architecture, LFA-1 is also an important co-stimulatory molecule during T cell activation by increasing the sensitivity for antigen by 100-fold. Mechanistically, LFA-1 engagement is known to enhance activation of early TCR signaling molecules and to promote later events of T cell proliferation and cytokine production (35, 36). LFA-1 plays a role also at earlier stages of synapse formation. During scanning in search of matching TCR/pMHC, the initial adhesive interactions between T cells and APC are mediated by LFA-1 and ICAM-1,3 on T cells and APCs, respectively (37). The functional relevance of LFA-1 on contact duration has been addressed by *in vivo* studies that correlated contact duration with acquisition of effector functions. Expression of the LFA-1 ligand ICAM-1 is required to sustain long antigen-specific DC–T contacts, whereas short interactions can still occur in the absence of ICAM-1. Importantly, T cells primed by ICAM-1 deficient DCs undergo early events of activation but fail to differentiate into effective memory CD8 T cells (38, 39). A mirroring finding in CD4 T cells lacking LFA-1 support is the importance of the LFA-1/ICAM-1 adhesion module to achieve optimal T cell priming *in vivo* (38). Interference with positive regulator of integrin activation yielded similar results. For instance, deletion of Talin in T cells leads to unstable contacts with APCs and failure to undergo full T cell activation (40, 41).

Soluble immunotransmitters like chemokines play also an important role in IS stabilization and T cell proliferation (42, 43). When approaching an APC, T cells emit CCR5 (or CXCR4)-enriched protrusions that indent the APC surface; this situation resembles the concentration of chemokine receptors at the leading edge of chemoattractant-stimulated T cells (44). These interactions culminate in the formation of a stable synapse, whereas CCR5 and CXCR4 are stably concentrated. Chemokine release at the immunological synapse and chemokine receptor recruitment into this region result in prolonged T-cell–APC interaction, and facilitate T cell activation by reinforcing T cell–APC pair attraction and delivering co-stimulatory signals (43). Interestingly, chemokine recognition in the context of the immunological synapse induces a *G*_q/11_-mediated CCR5 signaling, suggesting that chemokine receptor signaling pathways are modified by TCR triggering (43). Notably, coupling of *G*_q_ to the chemokine receptors delays their internalization, explaining the accumulation of CCR5 and CXCR4 at the T cell immunological synapse. In this scenario, chemokine receptors prolong the duration of T cell–APC interaction and facilitate T cell activation by increasing LFA-1 affinity (45), reinforcing T cell–APC pair attraction and avoiding pre-mature splitting due to other chemoattractant sources. On the basis of their actions, a dual role for chemokines in T-cell activation has been proposed, while the presence of chemoattractant forces when T cells are searching for the right partner may indeed prevent T cell–APC pairing, production of chemokines by the APCs, and subsequent accumulation and trapping of *G*_q_-coupled chemokine receptors at the IS, may represent a strategy to reinforce T-cell–APC interaction and facilitate T-cell activation (46, 47).

## The Wiskott–Aldrich Syndrome

Cytoskeletal remodeling is a highly dynamic process that ensures spatio-temporal coordination of diverse functions, such as mechanical support to the cell cortex, migration, phagocytosis, intercellular interactions, and subcellular distribution of signaling molecules and vesicles flow. Actin dynamics are tightly controlled by several different nucleation-promoting factors in turn activated by multiple complex pathways. Formation of branched actin networks is regulated by the Arp2/3 complex that is induced by the VCA domain contained in the WASp family of actin regulatory proteins. The eight members of the family (N-WASp, WAVE 1–3, WASH, JMY, and WHAM (48)] have different activation modes and control differential functions in various tissues. WASp, the founding member of the family, is expressed exclusively in the hematopoietic lineage and it was first discovered because loss-of-function mutations in its coding gene are associated with the X-linked immunodeficiency Wiskott–Aldrich syndrome (WAS) (49). The disease is characterized by multiple clinical manifestations, including susceptibility to infections, hemorrhages and eczema, and multiple forms of autoimmune disorders (50).

Wiskott–Aldrich syndrome protein mutations impact on disparate cellular functions in different hematopoietic lineages (51, 52). T cells were the first lineage recognized as being heavily affected by WASp mutations. A detailed review on the role of WASp in T cells has been recently published (53). Here, we will recall the main features of WASp deficient T cells and present the emerging defects in WASp null APCs.

Initial studies identified defects in TCR signaling and activation of IL-2 in T cells from WAS patients (54–56). WASp null T cells, similarly to cells of other hematopoietic lineages, also present with alteration of motility (57, 58). Later studies helped to better define how WASp controls selectively multiple sequential events in T cell activation. WASp is recruited to sites of early TCR receptor signaling in multimeric complex together with LAT, SLAP-76, Nck, and the cdc24 GEF Vav (32, 59, 60). At the synaptic interface, binding of activated cdc42, PIP2, and phosphorylation of tyrosine 291 by Src family kinases cooperate to release the auto-inhibited conformation of WASp, exposing the VCA domain and inducing acting nucleating activity [reviewed in Ref. (61)]. Genetic deletion of WASp in T cells causes alterations in the early dynamic events of stabilization of the synapse. Upon TCR triggering cycles of stable symmetric synapse structure alternates to phases of T cell motility when the synaptic structure is lost. WASp is required to reform the synaptic structure after these periodic breaking rather than for the initial synapse formation (62). This is in line with the finding that T cells derived from WASp patients, despite normal conjugate formation, fail to spatially organize signaling in the cSMAC and to polarize the microtubules organizing center (63). Downstream events of T cell activation, such as calcium fluxes, IL-2 production, and T cell proliferation, are also affected by WASp deficiency both in mouse models and in patient’s-derived cells (64–68). The exact role of WASp-mediated F-actin dynamics in regulating synaptic structure and downstream signaling is still not fully resolved. A recent study proposes that WASp controls selectively a small fraction of synaptic F-actin required to sustain PLC-γ activation and calcium ion elevation, thereby linking the control of early events to later T cell activation (69). It is also emerging that WASp can have actin-independent activities in T cells, functioning as a transcription factor to regulate transcription of cytokine genes (70). Thus, WASp plays a central role in controlling multiple integrated functions that link TCR signaling to full T cell activation. Moreover, its role varies depending on the T cell subset, reflecting the existence of cell type-specific modes of actin regulation besides common shared mechanism (53).

Regulated cytoskeletal remodeling is needed also to support the function of APCs during synapse formation and maintenance. DCs are active player in synapse formation by virtue of their membrane protrusions that facilitate scanning of the T cell repertoire and interaction with T cells (14, 71, 72). This flypaper membrane activity of DCs is regulated by members of the Rho family of small GTPase and by actin regulatory proteins. Genetic deletion of an upstream regulator of cytoskeletal remodeling, the Rho GTPases Rac, inhibits dendrites extension, resulting in reduced DC–T contact time and inefficient priming (71). In WAS, loss of proper actin cytoskeletal rearrangement hampers the function of DCs at several levels. Defects in adhesion to ICAM-1, polarization and responses to chemokine gradients (73, 74) render DCs unable to properly migrate from site of antigen acquisition in the periphery to lymph nodes. Failure to properly initiate adaptive immunity by WASp deficient DCs arises from defects that go beyond the capacity to properly home to lymph nodes (75). Delivery of the model antigen DEC205-OVA to resident DCs resulted in poor activation of antigen-specific CD8^+^ T cell in WASp null recipient. Further experiments to dissect the individual contribution of migration, antigen processing and DC–T cell interaction *in vivo* demonstrated that WASp null DCs fail to efficiently prime naïve CD8 T cells even when the migratory defect is compensated (75). Imaging of DC–T cell contacts *in vitro* and by two-photon microscopy *in vivo* indeed showed that WASp null DCs fail to form stable and long-lasting interactions with antigen-specific T cells (75). Interestingly, T cells primed by WASp null DCs can enter the cell cycle but fail to accumulate, similarly to what happens when priming is promoted by Cdc42 knock down DCs, an upstream regulator of WASp (76). A similar defect in the stability of DC–T cell contacts and in the capacity to support formation of an organized synaptic structure was seen also using CD4^+^ T cells (77). Taken together, these data indicate that defective cytoskeletal organization in WAS DCs affects two key steps during priming, i.e., migration to lymph nodes and formation of stable DC–T cell contacts and T cell activation once in lymph nodes. Thus, not only presentation of antigens that are taken up in the periphery and transported to secondary lymphoid organs but also presentation of blood born antigens by lymph node resident DCs is likely to be compromised in WAS. The impact of DCs to the overall immune deficiency is demonstrated by the fact that rescue of DCs functions upon gene therapy is capable to improve T cell priming (78).

It is also emerging that plasmacytoid DCs and myeloid cells present with defects in innate immunity pathways in WAS (79). The role that an altered cytokine secretion profile may have on synapse stability and signaling at the IS is an intriguing aspect that is currently being investigated by our group.

Wiskott–Aldrich syndrome patient experience recurrent autoimmune manifestations, whose cellular basis are not yet fully understood (80). Functional defects in regulatory T cells are likely to contribute to loss of peripheral tolerance (81–83). DC–T cell interactions are critical for the establishment of peripheral T tolerance besides initiation of adaptive immunity (17, 84). It is interesting to speculate that besides cell intrinsic Tregs abnormalities, defective interaction with APC may contribute to loss of peripheral tolerance in WAS.

## Other Actin-Related Immune Deficiencies

Recently, a new cytoskeletal-related immunodeficiency caused by mutation in the WASp interacting protein WIP has been identified (85). WIP controls WASp activity in at least three different ways: regulating its stability, controlling its activation by Cdc42, and bringing WASp to sites of active polymerization (86). Indeed, a stop codon mutation in the WIP sequence that silenced protein expression resulted in almost undetectable WASp level, and clinical features similar to WAS (85). At the cellular level, WIP was shown to control podosomes assembly and cell migration in DCs (87, 88). In addition, WIP binds to actin and controls cytoskeletal integrity independently of WASp. The WASp-independent actin regulation exerted by WIP is essential for T cell homing to infected tissue (89). A further interesting function that has been attributed to WIP, independently of its binding to WASp, is the control of lytic granule secretion in NK cells. The failure in cytolytic activity of WIP null NK cells is due to lack of transport and polarization of granules at the IS (90). The role of WIP in controlling IS formation in T cells and DCs has not yet been addressed. However, it is likely that priming will not be efficient because of defects on both sides of the IS, thus explaining the poor immune responses (91).

A further example of immunodeficiency arising form cytoskeletal abnormalities that affect synapse formation is DOCK8 deficiency. DOCK8 is a GTP-exchange factor for Rho and Rac GTPases that controls conversion of extracellular signals into activation of actin regulatory proteins. Mutations in DOCK8 were found to be the genetic basis of a combined immunodeficiency characterized by increased susceptibility to skin viral infections, hyper IgE syndrome, T cell lymphopenia, and impaired antibody response (92). At the cellular level, DOCK8 was shown to be required for the accumulation of the integrin ICAM-1 at the B cell synapse and its mutation compromise synaptic architecture and B cell functions (93). Marginal zone B cells are highly reduced in DOCK8, similarly to what has been observed in WAS (94). DOCK8 mutant T cells were also shown to have defective LFA-1 polarization in synapse, resulting in decreased T cell proliferation and survival (95). The DC compartment is also affected in a way reminiscent of defects observed in WAS, such as defective homing to lymph nodes and reduced T cell priming activity (96). Although the direct role of DOCK8 in controlling the stability of the DC-T synapse has not been addressed, it is reasonable to predict that alterations in contact duration may contribute to disease pathology.

## The WHIM Syndrome

As discussed above, chemokines and their receptors have a dual role in localization of T cells and APCs within secondary lymphoid organs, as well as in enhancing the strength of the T–APC interaction. Intriguingly, the relevance of the chemokine–chemokine receptor axis in promoting stable synapses has been further emphasized by recent studies on the rare immune deficiency warts, hypogammaglobulinemia, infections, and myelokathexis (WHIM). A chemokine-mediated regulation of the duration of T–APC interactions was shown to contribute to the cellular basis of T cell-dependent response defects in this disease (47). The WHIM syndrome is an inherited immunodeficiency that features a wide range of symptoms, including recurring infections, human papillomavirus (HPV)-induced warts, reduced long-term immunoglobulin G (IgG) titers, myelokathexis, and leukopenia (97–100). The syndrome is associated with dominant mutations in the chemokine receptor CXCR4 that lead to truncation of its carboxy-terminal domain. This leads to a defect in the ability of the receptor to internalize after binding its cognate ligand, CXCL12. As a consequence, immune cells bearing the WHIM-mutant receptor display increased signaling and enhanced migration after stimulation by chemokine (98, 101–103). Historically, this enhanced functionality of the mutant CXCR4 has provided a mechanistic explanation for the abnormal retention of neutrophils in the bone marrow (myelokathexis), as demonstrated by experiments in a human-to-mouse *in vivo* xenograft model and in a zebrafish model (101, 104). Yet, symptoms of WHIM syndrome patients, such as the inability to successfully mount responses to a recurring pathogen and the decreased capacity to produce hypermutated IgG signify that antigen-specific memory responses, antibody class-switching and affinity maturation are defective in these individuals (99). The finding that CXCR4, along with other chemokines, is utilized in the organization of lymphoid organ follicles enabled the speculation that possible aberrations in lymphoid organ architecture could be the cause of the above adaptive immunity defects in WHIM (99). Reports of disrupted lymph node spatial organization in a recent mouse knock-in model of WHIM support this hypothesis (105). Nonetheless, the generation of antigen-specific memory, Ig class switching and affinity maturation do depend on the formation of successful T cell–APC interactions via immunological synapses (106). Recent work has identified that T cells from WHIM patients, or indeed healthy donor T cells transfected with the dominant, WHIM-mutant CXCR4, form less stable conjugates with superantigen-pulsed B cells. Importantly, this only occurs in the presence of competing migratory (“go”) signals from exogenous CXCL12, which appear to affect the mutant but not the wild-type receptor. A very similar impairment of T–APC immunological synapse stability occurs between antigen-specific WHIM-mutant T cells and antigen-loaded DCs in *ex vivo* lymph node slice cultures derived from a retrogenic model of WHIM, imaged via 2-photon microscopy (47). While both wild-type and WHIM-mutant CXCR4 are recruited to the immunological synapse, exogenous CXCL12, which is present in lymph nodes (107), is able to “distract” only the hyperfunctional WHIM-mutant CXCR4 away from the synapse. Indeed, wild-type CXCR4 is unable to impair immunological synapse formation (108) and has no effect on T cell activation (109, 110). Intriguingly, however, the hyperfunctional WHIM-mutant CXCR4 appears to exceed a threshold that favors motility over formation of stable immunological synapses, resulting in aberrant T cell activation (47). Further molecular studies will tell us more about the regulation of T–APC interactions. Nonetheless, the finding that many of the WHIM defects are reversible using a pharmacological inhibitor of CXCR4 is an interesting demonstration of how chemokines and their receptors, in specific circumstances, have the ability to affect T cell function.

## Synaptic Defects in Patients with X-Linked Lymphoproliferative Disease

X-linked lymphoproliferative disease (XLP) is caused by loss-of-function mutations in signaling lymphocyte activation molecule-associated protein (SAP), an adaptor linking SLAM family receptors to downstream signaling. The protein is primarily expressed in T cells, NK cells, and B cells. XLP patients are subjected to severe Epstein–Barr viral infections and develop lymphomas and lymphoproliferative disorders (111). At the cellular level, the disease is characterized by a defect in germinal center formation and consequently poor humoral response, abnormalities in NKT cell development, NK cell cytotoxicity, and cytokine production (112). In the context of this review, it is interesting to discuss the evidences pointing to disturbed B–T cell interaction to explain poor germinal center formation. Upon initial activation of T cells by DCs in lymph nodes, the second circuit of immune cell interaction includes motile but prolonged interactions between activated B cells and T cells at the border between the follicle and the T cell zone, followed by translocation of T cells in the germinal center to sustain the germinal center reaction. Follicular helper T cells, specialized in this process, express high levels of SAP and SLAM (113). *In vivo* imaging of B–T interactions during T cell-dependent B cell-activation revealed that SAP-deficient T cells are intrinsically unable to form stable contact with B cells. Interestingly, this defect is selective for B–T cell interaction, as DC–T cell interactions proceed normally. These data show that SAP-associated family members controls, selectively, adhesive mechanism required to stabilize T cell–B cell conjugates required to deliver to B cells the signals supporting full B cell proliferation (112, 114). Further insight into the role of SAP and SLAM receptor in assembling B–T synapse comes from the finding that the SLAM receptor Ly108 is a potent negative regulator of T–B cell adhesion, counteracted by SAP, that act by recruiting the phosphatase SHP-1 at the synapse (115).

SAP functions also in controlling adhesion during cytolysis. SAP-deficient cytotoxic T lymphocytes fail to assemble a proper synaptic structure during conjugation to target cells, with altered polarization of perforin granules and lipid raft at the contact site (116, 117). In line with this observation, SAP-deficient NKT cells fail to polarize the microtubule-organizing center toward the target cell, resulting in reduced killing ability (118).

## Conclusion

A class of primary immunodeficiency is caused by pathogenic mutations in genes controlling immune cell trafficking and cellular interactions dynamics (Figure 1). The cellular basis of these diseases has been increasingly investigated helping to improve patients management. Moreover, analysis of these naturally aris­ing mutant cells revealed important insights into basic functioning of the immune system. As a prominent example, WASp mutant cells have been instrumental in understanding actin-mediated signal transduction during TCR triggering and to unveil the importance of an intact actin cytoskeleton in APCs. Characterization of mutant T cells in less common immunodeficiency like WHIM, WIP, DOCK8, and SAP is still at early stages and it will help to dissect subtle details of immune cell interaction regulation. Further analysis is needed to understand the reciprocal contribution of alterations on both sides of the IS to gain an integrated view of the parameters that control, in normal and pathological conditions, the transfer of information between APC and T cells during priming of adaptive immune responses.

## Conflict of Interest Statement

The authors declare that the research was conducted in the absence of any commercial or financial relationships that could be construed as a potential conflict of interest.
